# Fatigue Analysis of a 40 ft LNG ISO Tank Container

**DOI:** 10.3390/ma16010428

**Published:** 2023-01-02

**Authors:** Du-Yong Lee, Jae-Sang Jo, Antony John Nyongesa, Won-Ju Lee

**Affiliations:** 1 Korea Marine Equipment Research Institute, Busan 46754, Republic of Korea; 2 Division of Marine Engineering, Korea Maritime and Ocean University, Busan 49112, Republic of Korea; 3 Department of Mechanical IT Convergence Engineering, Korea Maritime and Ocean University, Busan 49112, Republic of Korea; 4 Interdisciplinary Major of Maritime AI Convergence, Korea Maritime and Ocean University, Busan 49112, Republic of Korea; 5 Division of Marine System Engineering, Korea Maritime and Ocean University, Busan 49112, Republic of Korea

**Keywords:** LNG, finite element analysis (FEA), ISO tank, Ansys Mechanical

## Abstract

The demand for Liquefied natural gas (LNG) has rapidly increased over the past few years. This is because of increasingly stringent environmental regulations to curb harmful emissions from fossil fuels. LNG is one of the clean energy sources that has attracted a great deal of research. In the Republic of Korea, the use of LNG has been implemented in various sectors, including public transport buses, domestic applications, power generation, and in huge marine engines. Therefore, a proper, flexible, and safe transport system should be put in place to meet the high demand. In this work, finite element analysis (FEA) was performed on a domestically developed 40 ft ISO LNG tank using Ansys Mechanical software under low- and high-cycle conditions. The results showed that the fatigue damage factor for all the test cases was much lower than 1. The maximum principal stress generated in the 40 ft LNG ISO tank container did not exceed the yield strength of the calculated material (carbon steel). Maximum principal stress of 123.2 MPa and 107.61 MPa was obtained with low-cycle and high-cycle analysis, respectively, which is 50.28% less than the yield strength of carbon steel. The total number of cycles was greater than the total number of design cycles, and the 40 ft LNG ISO tank container was satisfied with a fatigue life of 20 years.

## 1. Introduction

LNG fuel has attracted a great deal of research attention as a potential source of clean energy. Natural gas, which is obtained from the boiling of vapor of LNG, is mainly made up of methane (CH4) which is the simplest hydrocarbon compound [1]. It has only one carbon atom in its chemical structure. Research on LNG usage has revealed a significant reduction in carbon-based exhaust gas emissions, especially in internal (IC) combustion engines [2,3,4,5]. With increasingly tight environmental regulations, LNG fuel is a potential clean fossil fuel. In the Republic of Korea, LNG usage has been implemented in various sectors, including city gas pipelines supplied to each household and public transport buses. Gradually, LNG is being used in various industries such as power generation and shipbuilding. In recent news, the Republic of Korea also plans to replace the existing ships with LNG-fueled ships [6]. Therefore, SK Gas, which is the largest gas producer in the country, has invested heavily in the expansion of LNG infrastructure. Compared with diesel or heavy fuel oil, LNG has a higher lower calorific value, and therefore an IC engine operating at the same power output will require a smaller amount of LNG fuel [1]. As the demand for LNG increases, research and development for a method of supplying LNG is also being actively carried out [7,8,9,10,11,12,13]. The method of supplying NG (natural gas) to consumers can be generally divided into ‘NG supply through the piping network’, ‘LNG supply through tank lorry’, and ‘LNG supply through tank container’.

**Figure 1:**
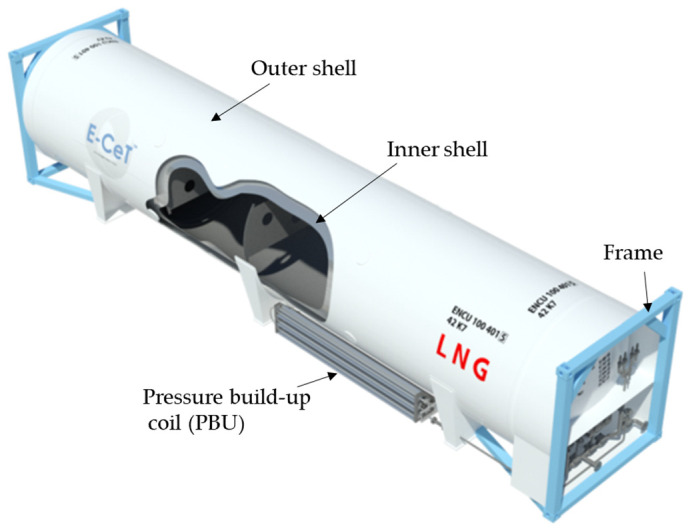
40 ft LNG ISO tank.

An ISO tank container is a liquid container manufactured according to internationally agreed standards such as the International Maritime Organization (IMO) and International Organization for Standardization (ISO). They are used for transporting large volumes of liquids, door to door, safely and efficiently. They comprise an inner tank made of stainless steel that can store liquid at −196 °C and an outer tank made of carbon steel to which vacuum insulation is applied for insulation, and the inner tank is assembled in the outer tank. Previously, the Republic of Korea depended on imported ISO tanks from countries such as China for use domestically. However, with the increasing demand for LNG worldwide it is very competitive, expensive, and difficult to acquire enough ISO tanks to sustain the Republic of Korea’s fast-growing demand for LNG. For these reasons, local manufacturers decided to develop ISO tanks within the country. Figure 1 illustrates a newly developed LNG 40 ft. ISO tank.

During the development process of ISO tanks, cranes are used to manually assemble the inner and the outer shells. Manually assembled shells have the disadvantage of non-uniform space between the shells ranging from 100 to 300 mm. Varying spaces between the shells limits the load carrying capacity of the ISO tank. In this study, the newly designed 40 ft. ISO tank was assembled using a special patented process to make a constant space of 100 mm between the inner and outer shell. A constant space between the shells enables a higher warm full volume of 44.043 m^3^ (approximately 18 tones), which is 1.78% higher than existing LNG ISO tanks when filled up to 90% as stated in the international safety standards. ISO tanks are an important component for LNG transportation, especially with the growing demand for cleaner energy sources; however, progress in research and development of ISO tanks is not available. The authors could not find related literature. This study has mentioned the design improvement (higher carrying capacity) in the ISO tank used in this study; however, detailed information is out of the scope of this study. This study will only focus on fatigue analysis at low- and high-cycle conditions.

Finite element analysis (FEA) using ANSYS simulation software is a well-known tool that has been successfully used by many researchers and yielded excellent results. Ganeshkumar et al. [14] conducted an FEA using ANSYS to investigate the tensile properties of different infill pattern structures of 3D-printed polymers and found that hexagonal patterns held remarkable mechanical properties. Agrawal et al. [15] also used ANSYS to perform a finite element stress analysis for shape optimization of spur gear. They managed to develop an optimized spur gear with a reduced maximum stress of up to 12.93%. Fahy et al. [7] conducted an FEA of a container tank to model the tank and the frames using ANSYS 5.4. They researched dynamic and static conditions. They validated their results using the ISO tests conducted by Companie Auxilliere Industrie Belgique (CAIB). Their results showed a good agreement with the experimental results. Wang et al. [16] used ANSYS software to conduct an FEA of an LNG tank container for trains under inertial force. They analyzed the stress and strength under five load cases. The results showed that the Mises equivalent membrane stress at the frame is controlled by the inertial force but at the inner vessel, it depends on the internal pressure, although it may be significantly affected by the inertial force in some cases. Cao et al. [17] used the finite element method to analyze the influence of the liquid inertial force on the stress distribution at the frame and shell of tank containers under different loading modes and obtained very good results. Other related research can be found in [18,19,20].

The success of ANSYS software in previous research works encouraged the authors to use similar software in this study. The current work employed an FEA method to predict the fatigue life of a domestically developed 40 ft ISO LNG tank with an improved maximum carrying capacity. The study was conducted using Ansys Mechanical software under low- and high-cycle loading conditions. The result showed that the maximum stress generated in the 40 ft LNG ISO tank container did not exceed the yield strength of stainless steel and carbon steel. Maximum principal stress of 123.2 MPa and 107.61 MPa was obtained with low-cycle and high-cycle analysis, respectively, which is 50.28% less than the yield strength of carbon steel. The total number of cycles was greater than the total number of design cycles, and the 40 ft LNG ISO tank container was satisfied with a fatigue life of 20 years. It is worth noting that, even though the demand for LNG is growing, LNG ISO tank-related literature is very limited. This study highlights the research progress in LNG transportation and fatigue analysis, which is currently rare.

## 2. Materials and Methods

### 2.1. Computational Domain and Materials

**Figure 2:**
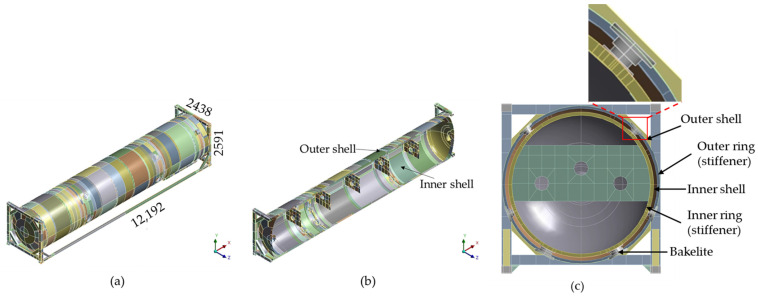
(**a**) Computational domain with dimensions (mm); (**b**) longitudinal section; and (**c**) transverse section of the computational domain.

The 3D computational geometry of the 40 ft. LNG ISO tank was generated using the Ansys SpaceClaim tool provided by the ANSYS workbench. The geometry comprises the inner vessel, vessel support, outer jacket, and frame structures. The model geometry was drawn with similar dimensions as the actual tank. Figure 2 shows (a) 3D computational geometry with dimension (mm) and (b) half geometry showing interior space.

The inner shell and head, baffle plate, and baffle supporting ring are assigned stainless steel (SA240-304), while carbon steel (A36) is applied to the outer shell and the head. The holder is assigned Bakelite material similar to the material of the actual ISO tank. The selection of material was done as per the requirements stated in the ASME sections II and VII. Table 1 and Table 2 show the properties of stainless steel and carbon steel, respectively.

### 2.2. Computational Mesh

**Figure 3:**
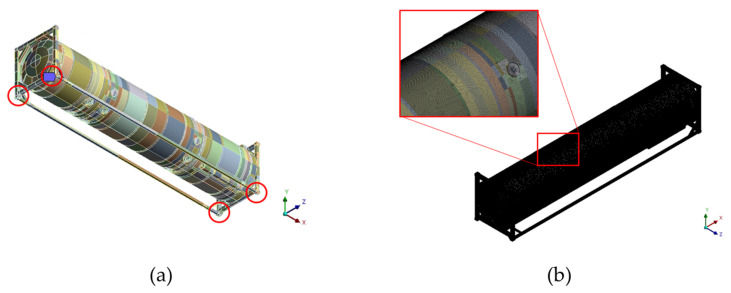
(**a**) Constrained positions of the computational domain. (**b**) Computational mesh.

FEA involves two major steps, namely establishment of governing equations and solving governing equations. Meshing allows the division of a complex geometry into simpler shapes called finite elements. In this study, the FEA was conducted for high and low-cycle conditions. ISO tanks are subjected to different forces and stresses during operation. Therefore, the international standard ISO 1496-3 part 3 outlines testing procedures for tank containers used for carrying liquids, gases, and pressurized dry bulk. This series of tests ensures that the designed tank can withstand resultant stress during its lifetime. Low cycle refers to the test condition when the testing tank is in a stationary condition, while high-cycle condition refers to the test condition when the tank is in transit either by road, rail or by sea. Low-cycle condition was achieved by constraining the degree of freedom. In 3D structural analysis, the elements must have six degrees. This means that the elements can move under translation in x, y, and z directions and can also rotate about x, y, and z directions. Therefore, the 3D computational domain was constrained at the bottom casting area in the x, y, and z direction ends as shown by red circles in Figure 3a. Constraining the degrees of freedom creates boundary conditions and it also makes modeling possible [22]. The high-cycle condition was achieved by applying accelerations to the three corners of the ISO tank in separate cases of the simulation. The meshing of the 3D computational domain was performed using the Ansys meshing tool. The selection of a suitable mesh directly affected the accuracy of the results and the computational time. The computational mesh was composed of tetrahedron mesh consisting of 867,585 elements and 777,067 nodes. The mesh quality was of great importance during the study. A mesh with high quality was generated for calculations. The mesh quality of a minimum orthogonal ratio of 0.152, maximum skewness of 0.8479, and a minimum aspect ratio of 1.0822 was used. These mesh characteristics lie within the recommended range provided by ANSYS [22] for a finite element simulations. Figure 3b shows a computational mesh.

### 2.3. Boundary Conditions

**Figure 4:**
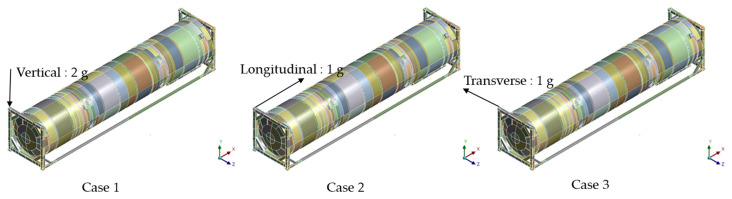
Illustrations of the acceleration locations during high-cycle loading conditions for simulation cases 1, 2 and 3.

Analysis was conducted at high-cycle and low-cycle conditions. For the low-cycle test case, the computational domain was constrained, as shown in Figure 3a. The operating condition of the inner tank pressure was set at 0.509 MPa, while the outer shell was set at 0.101 MPa. The dead weight was set at 30,480 kg. During the high-cycle tests, an acceleration of 2 g was applied in the vertical direction (x-axis), a 1 g acceleration was applied to the longitudinal direction (y-axis), and a 1 g acceleration was applied in the transverse direction (z-axis) according to ISO 1496-3. The accelerations were applied separately to form 3 high-cycle simulation cases. Figure 4 shows the acceleration application position and direction during cases 1, 2, and 3, respectively. The FEA in this case involved mechanical loads; it was solved using a direct coupled approach in which mechanical matrices were solved in parallel.

## 3. Results

### 3.1. Maximum Principal Stress

The study on fatigue analysis of materials can be achieved using three approaches. They include the fracture mechanics method, strain life method, and stress life method [23]. The third method was applied in this study; therefore, analysis of the maximum principal stress was necessary to correctly determine the fatigue life of the ISO tank.

**Figure 5:**
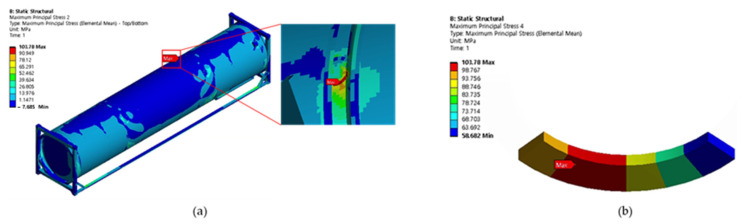
Case 1 principal stress distribution. (**a**) Entire computational domain showing region of maximum stress (position 1). (**b**) Position 1 element analysis result in detail.

**Figure 6:**
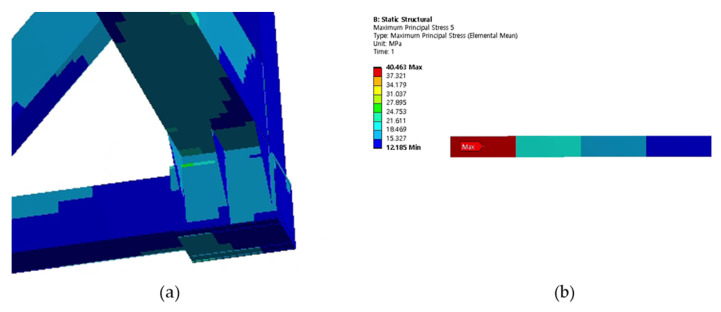
Case 1 principal stress distribution. (**a**) Entire computational domain showing region of maximum stress (position 2). (**b**) Position 2 element analysis result in detail.

**Figure 7:**
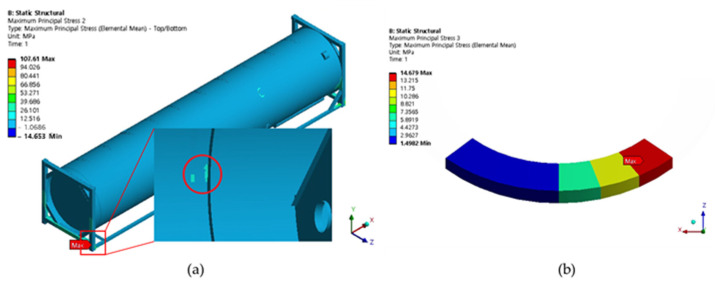
Case 2 principal stress distribution. (**a**) Entire computational domain showing region of maximum stress (position 1). (**b**) Position 1 element analysis result in detail.

**Figure 8:**
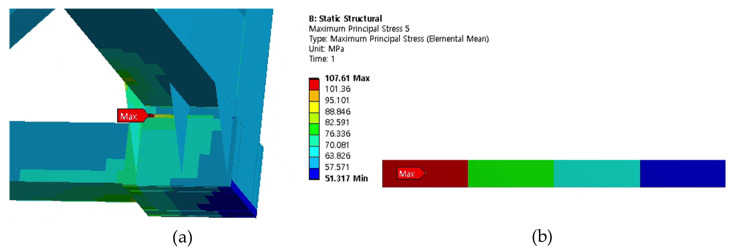
Case 2 principal stress distribution. (**a**) Entire computational domain showing region of maximum stress (position 2). (**b**) Position 2 element analysis result in detail.

**Figure 9:**
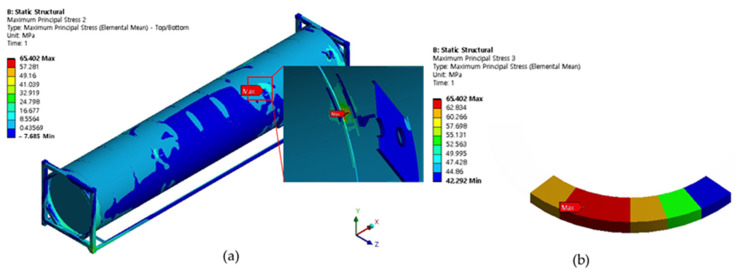
Case 3 principal stress distribution. (**a**) Entire computational domain showing region of maximum stress (position 1). (**b**) Position 1 element analysis result in detail.

**Figure 10:**
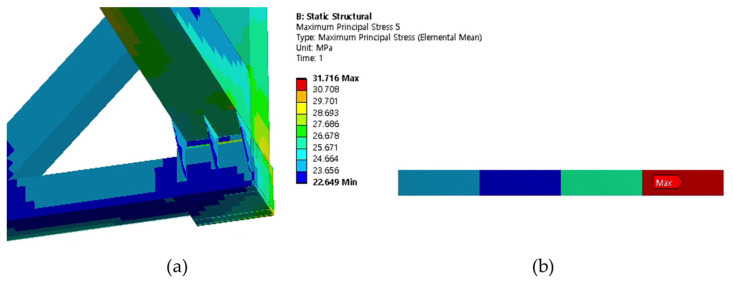
Case 3 principal stress distribution. (**a**) Entire computational domain showing region of maximum stress (position 2). (**b**) Position 2 element analysis result in detail.

**Figure 11:**
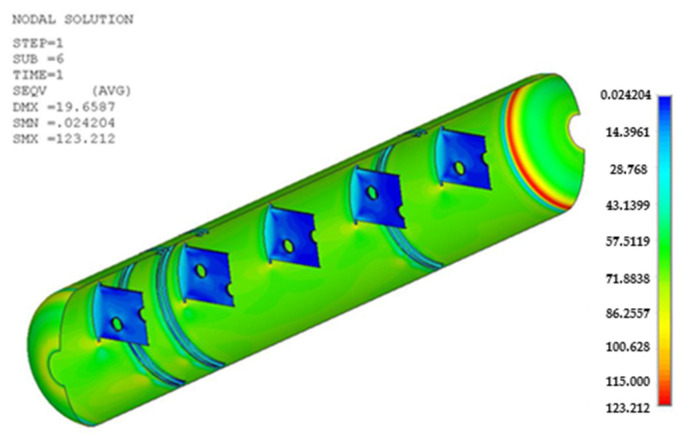
Case 4 principal stress contour map distribution results for low-cycle fatigue analysis.

Cases 1, 2, and 3 represent the high-cycle analysis, while case 4 is the low-cycle analysis. In each case, the maximum principal stress locations are defined as the failure positions. Therefore, two points (position 1 and position 2) with maximum principal stresses were determined in each case and later were used for fatigue analysis. The inner and outer shells were connected by Bakelite. When the inner shell was filled with LNG, the weight was applied to the lower Bakelite and had an effect. There were weldments used to connect various parts of the ISO tank; however, the first vulnerable part was around the Bakelite that supports the inner and outer stiffener in the lower region of the tank, and the second vulnerable part was the container lashing part. Although both parts are vulnerable, analysis results show that the designed tank can achieve a design life of 20 years. The summary results of the maximum principal stress at the failure positions are shown in Table 3. The stress contour distribution showing maximum principal stress locations for high-cycle analysis is shown in Figure 5, Figure 6, Figure 7, Figure 8, Figure 9 and Figure 10, while low-cycle stress contour map distribution results are shown in Figure 11.

### 3.2. Fatigue Analysis

The results for the maximum principal stress obtained from the FEA simulations were used to perform fatigue analysis on the LNG 40 ft ISO container. Fatigue analysis was conducted based on ASME code section VIII, Division 2, part 5 (5.5.3.2). This code provides a detailed procedure for fatigue assessment basing on the elastic stress and equivalent stresses. All formulas stated in this section can be found in the literature [24]. According to the design data, the ISO tank has a design number of cycles of 10^8^ cycles for cyclic temperature and pressure cycles considering a design life of 20 years. The determination of the effective alternating equivalent stress amplitude for the number of cycles is given by Equation (1).

(1) $$S_{alt,k}=\frac{K_{f}\cdot K_{e,k}\cdot \left(\Delta S_{p,k}-\Delta S_{LT,k}\right)+K_{v,k}\cdot \Delta S_{LT,k}}{2}\;$$

However, in this study, the fatigue penalty factor $K_{e,k}$ was used for the entire stress range. In this case, ASME code states that values of the Poisson correction factor $K_{v,k}$ and $\Delta S_{LT,k}$ can be omitted. Therefore, the formula becomes:

(2) $$S_{alt,k}=\frac{K_{f}\cdot K_{e,k}\cdot \Delta S_{p,k}}{2}=S_{a}$$

In Equation (3), $S_{a}$ is the computed stress amplitude, $K_{f}$ = 1.2 is the fatigue strength reduction factor obtained from Table 5.11 of the ASME VIII code division 2. $K_{e,k}$ is the fatigue penalty factor and it is equal to 1 since $\Delta S_{p,k}<S_{PS}$. $\Delta S_{p,k}$ is the component stress range.

**Figure 12:**
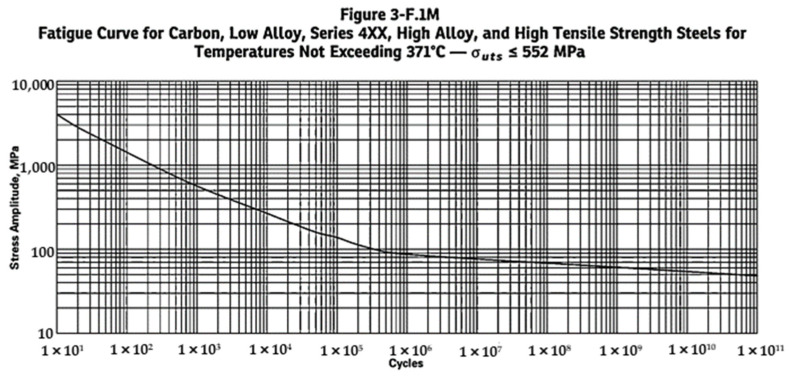
Fatigue curve for carbon, low alloy, series 4XX, high alloy and high tensile strength steels for temperatures not exceeding 371 °C [25].

The permissible number of cycles N is then determined for the equivalent stress computed in Equation (2) using the fatigue curves based on the materials of construction provided in annex 3-F, 3-F.1 of the ASME VIII code (Figure 12).

The exponent used to determine the permissible number of cycles (*X*) is computed using the stress factor (*Y*). For $10^{y}<20$ *X* is computed as

(3) $$X=\frac{38.1309-60.1705Y^{2}+25.0352Y^{4}}{1+1.80224Y^{2}-4.68904Y^{4}+2.26536Y^{6}}\;$$

While for *X* ≥ 20

(4) $$X=-4706.5245+1813.6228Y+\frac{6785.5644}{Y}-368.12404Y^{2}-\frac{5133.7345}{Y^{2}}+30.708204Y^{3}+\frac{1596.1916}{Y^{3}}\;\;$$

where

(5) $$Y=log\left[28.3\times 10^{3}\left(\frac{S_{a}}{E_{T}}\right)\right]\;$$

and $E_{T}$ is the modulus elasticity of the material.

The fatigue damage was determined by dividing the total number of the design cycles (*n*) by the permissible number of cycles (*N*) as shown in Equation (6).

(6) $$D_{f}=\frac{n}{N}$$

There were four cases analyzed in this study, of which three cases involved the high cycle and one case the low cycle. The maximum stress and other required parameters for fatigue analysis of both high- and low-cycle assessment and calculations were obtained from the FEA simulation and the recommended values in the ASME VIII Division 2 code. Using the formulas highlighted in the previous section Equations (1)–(6), the fatigue life prediction of the 40 ft LNG tank was carried out. Calculations for the fatigue damage factor, which determines whether the designed tank satisfies the intended fatigue life were as follows. In this paper, the calculations for high cycle, position one is shown. Similar calculation procedures were performed for the other study cases 2–4 and the results are presented in summary in Table 4.

There are 10^8^ cycles for cyclic temperature and pressure cycle, considering 20 years of design life. From the FEA, maximum and minimum stress values were determined. The component stress range $\Delta S_{p,k}$ for high-cycle case 1 at position 1 was 103.78 Mpa. The fatigue penalty factor was 1 since $\Delta S_{p,k}<S_{PS}$. The fatigue strength reduction factor was 1.2 as obtained from recommended values in Table 5.11 of the ASME VIII code division 2 [25]. Stress amplitude $S_{a}$ was computed by substituting values in Equation (2), which resulted in 62.27 Mpa. The stress amplitude and the modulus of elasticity was substituted in Equation (5) for *Y* (stress factor used to compute *X*). After solving the Equation (5), the result was 0.960 Mpa. Now using the determined stress factor used to compute *X* (exponent used to compute the permissible number of cycles), we determined the value of *X* using Equation (3) as 8.719. The design number of design cycles (*N*) is given as 10^X^ and previously, the value of *X* was determined as 8.719. Therefore, the number of design cycles can be written as 10^8.719^ (523,017,667.3 cycles). Previously, the total number of design cycle was found to be 10^8^, and using this value we could substitute in Equation (6) to get the fatigue damage factor $D_{f}$. In this case, the value of $D_{f}$. was found to be 0.191. The ratio of the number of cycles at a given stress level to the fatigue life is also known as cycle ratio. Practically, this relationship states that the fatigue failure occurs when the sum of the cycle ratios equals one. This relationship was proposed by A. Palmgren [26] for predicting the life of ball bearings and it was later applied in more general context by B. F. Langer [27]. The rule was not widely known until its appearance in the work of M. A. Miner [28]. The results of FEA of the 40 ft. LNG ISO tank show that the value of $\;D_{f}$ was much lower than 1.

## 4. Conclusions

This study was limited to the analysis of the fatigue life of a 40 ft LNG ISO tank under low-cycle and high-cycle conditions. Despite the fast-growing demand for LNG, research regarding its transportation using ISO tanks is very limited. The authors could not find any published literature related to this topic. The ISO tank used in this study is an improved design with a higher carrying capacity. When filled to 90%, the 40 ft ISO tank weighs about 18 tons, and because of this, problems due to stress concentration arise. Stress concentration occurs due to structural discontinuities, local deformation of welds, misalignment of weld joints, and welding defects, and occurs in welds between plates of different thicknesses and fillet welds in pipe fittings. Therefore, in order to reduce the fatigue intensity due to these stress concentrations, it is necessary to predict the fatigue life. The analysis was based on a design life consideration of 20 years. The fatigue damage factor (FDF) of the 40 ft LNG ISO tank container was determined for each case of the study. Maximum principal stress of 123.2 MPa and 107.61 MPa was obtained with low-cycle and high-cycle analysis, respectively, which is 50.28% less than the yield strength of carbon steel. For all the cases analyzed, the value of the FDF was found to be very small. At high-cycle FEA, the highest FDF value of 0.191 was obtained after applying an acceleration of 2 g vertically at position 1. When a longitudinal acceleration of 1 g was applied to the ISO container tank, an FDF value of 0.181 was found at position 2. In all the remaining cases considered in this study, FDF was found to be 0 for different positions and load conditions. In FEA, the critical FDF value is unity (1). The analyzed cases revealed that the designed tank is capable of achieving a design life of 20 years since all the values of FDF are very small and much less than unity. The results show that the designed 40 ft. LNG ISO tank is safe and capable of withstanding the designed fatigue life. Once commercialized, it will play a very important role in meeting the growing demand for LNG in different sectors, both domestically and internationally.

## Figures and Tables

**Table 1:** Properties of stainless steel [21].

Temperature (°C)	−196	−138	10	50
Thermal conductivity (W/mC)	14.8	14.8	14.8	15.3
Thermal expansion coefficient (1/K)	15.3	15.3	15.3	15.6
Young’s modulus (MPa)	208,700	204,900	197,100	193,000
Poisson’s ratio	0.31	0.31	0.31	0.31
Tensile strength (MPa)	515	515	515	515
Yield strength (MPa)	138	138	138	138
Density (kg/m^3^)	8030	8030	8030	8030

**Table 2:** Properties of carbon steel [21].

Temperature (°C)	−196	−138	10	50
Thermal conductivity (W/mC)	60.4	60.4	60.4	59.8
Thermal expansion coefficient (1/K)	11.5	11.5	11.5	11.8
Young’s modulus (MPa)	215,800	212,700	204,500	200,700
Poisson’s ratio	0.3	0.3	0.3	0.3
Tensile strength (MPa)	400	400	400	400
Yield strength (MPa)	248	248	248	240.5
Density (kg/m^3^)	7750	7750	7750	7750

**Table 3:** Maximum principal stress.

Load Case		Maximum Principal Stress (MPa)
Case 1	Position 1	103.78
	Position 2	40.463
Case 2	Position 1	14.679
	Position 2	107.61
Case 3	Position 1	65.402
	Position 2	31.716
Case 4	Position 1	123.2

**Table 4:** Results for the finite element analysis.

Parameter	Case 1		Case 2		Case 3		Case 4
	Pos. 1	Pos. 2	Pos. 1	Pos. 2	Pos. 1	Pos. 2	Pos. 1
$\mathrm{Fatigue}\;\mathrm{penalty}\;\mathrm{factor}\;K_{e,k}$	1	1	1	1	1	1	1
$\mathrm{Fatigue}\;\mathrm{strength}\;\mathrm{reduction}\;\mathrm{factor}\;K_{f}$	1.2	1.2	1.2	1.2	1.2	1.2	1.2
The component stress range $\Delta S_{p,k}$ [MPa]	103.78	40.463	14.679	107.61	65.402	31.716	123.2
$\mathrm{Computed}\;\mathrm{stress}\;\mathrm{amplitude}\;S_{a}$ [MPa]	62.27	24.28	8.81	64.57	39.24	19.03	73.92
$\mathrm{Modulus}\;\mathrm{of}\;\mathrm{elasticity}\;E_{T}$ [MPa]	193,000	200,700	193,000	200,700	193,000	200,700	193,000
Stress factor Y [MPa]	0.960	0.534	0.111	0.959	0.760	0.429	1.035
Exponent for permissible No. of cycles X	8.719	19.398	36.604	8.744	12.845	23.523	7.184
The permissible design cycles N	$5.2\times 10^{9}$	$2.5\times 10^{20}$	$4.02\times 10^{38}$	$5.5\times 10^{8}$	$7.0\times 10^{12}$	$3.3\times 10^{23}$	$1.53\times 10^{8}$
$\mathrm{The}\;\mathrm{total}\;\mathrm{number}\;\mathrm{of}\;\mathrm{design}\;\mathrm{cycles}\;n_{k}$	$10^{8}$	$10^{8}$	$10^{8}$	$10^{8}$	$10^{8}$	$10^{8}$	2000
$\mathrm{The}\;\mathrm{fatigue}\;\mathrm{damage}\;\mathrm{factor}\;D_{f,k}$	0.191	0.000	0.000	0.181	0.000	0.000	0.000

## Data Availability

Not applicable.

## Nomenclature

Symbols and Abbreviations	Meaning
$S_{alt,k}$	effective alternating equivalent stress amplitude
$K_{f}$	fatigue strength reduction factor
$K_{e,k}$	fatigue penalty factor
$\Delta S_{p,k}$	component stress range
$\Delta S_{LT,k}$	the component stress range for different the fatigue penalty factor
$S_{PS}$	maximum allowable stress
$K_{v,k}$	Poisson correction factor
$S_{a}$	computed stress amplitude
$E_{T}$	modulus elasticity of the material
n	total number of the design cycles
N	the permissible number of cycles
$D_{f}$	the fatigue damage factor
*Y*	stress factor used to compute X
*X*	exponent used to compute the permissible number of cycles
LNG	liquefied natural gas
ASME	American society of mechanical engineers
FDF	fatigue damage factor
